# Imbalance of Pulmonary Microvascular Endothelial Cell-Expression of Metalloproteinases and Their Endogenous Inhibitors Promotes Septic Barrier Dysfunction †

**DOI:** 10.3390/ijms24097875

**Published:** 2023-04-26

**Authors:** Devika P. Jayawardena, Marcello G. Masciantonio, Lefeng Wang, Sanjay Mehta, Natalie DeGurse, Cynthia Pape, Sean E. Gill

**Affiliations:** 1 Centre for Critical Illness Research, Lawson Health Research Institute, London, ON N6A 5W9, Canada; 2 Department of Physiology and Pharmacology, Schulich School of Medicine and Dentistry, Western University, London, ON N6A 3K7, Canada; 3 Division of Respirology, Schulich School of Medicine and Dentistry, Western University, London, ON N6A 3K7, Canada; 4 Department of Medicine, Schulich School of Medicine and Dentistry, Western University, London, ON N6A 3K7, Canada

**Keywords:** sepsis, PMVEC, metalloproteinases, TIMP, adherens junction, tight junction, synthetic metalloproteinase inhibitors, permeability

## Abstract

Sepsis is a life-threatening disease characterized by excessive inflammation leading to organ dysfunction. During sepsis, pulmonary microvascular endothelial cells (PMVEC) lose barrier function associated with inter-PMVEC junction disruption. Matrix metalloproteinases (MMP) and a disintegrin and metalloproteinases (ADAM), which are regulated by tissue inhibitors of metalloproteinases (TIMPs), can cleave cell–cell junctional proteins, suggesting a role in PMVEC barrier dysfunction. We hypothesize that septic PMVEC barrier dysfunction is due to a disruption in the balance between PMVEC-specific metalloproteinases and TIMPs leading to increased metalloproteinase activity. The effects of sepsis on TIMPs and metalloproteinases were assessed ex vivo in PMVEC from healthy (sham) and septic (cecal ligation and perforation) mice, as well as in vitro in isolated PMVEC stimulated with cytomix, lipopolysaccharide (LPS), and cytomix + LPS vs. PBS. PMVEC had high basal *Timp* expression and lower metalloproteinase expression, and septic stimulation shifted expression in favour of metalloproteinases. Septic stimulation increased MMP13 and ADAM17 activity associated with a loss of inter-PMVEC junctional proteins and barrier dysfunction, which was rescued by treatment with metalloproteinase inhibitors. Collectively, our studies support a role for metalloproteinase–TIMP imbalance in septic PMVEC barrier dysfunction, and suggest that inhibition of specific metalloproteinases may be a therapeutic avenue for septic patients.

## 1. Introduction

Sepsis is defined as life-threatening organ dysfunction caused by a dysregulated host response to infection [1]. Septic organ dysfunction is characterized by the activation and dysfunction of microvascular endothelial cells (MVEC), leading to a loss of microvascular barrier function [2]. For example, septic loss of barrier function of pulmonary MVEC (PMVEC) results in increased pulmonary microvascular permeability, which ultimately leads to severe pulmonary edema and acute respiratory distress syndrome (ARDS) [3]. While many mechanisms contributing to septic PMVEC injury and barrier dysfunction have been described, no treatment for the septic tissue damage and organ dysfunction has been identified [4,5,6,7,8,9,10,11,12]. Moreover, endogenous mechanisms protecting against sepsis-induced PMVEC barrier dysfunction are poorly characterized. One of these potential protective endogenous mechanisms is the balance between a family of enzymes, the metalloproteinases, and their inhibitors, the tissue inhibitors of metalloproteinases (TIMPs).

Metalloproteinases are a diverse group of endopeptidases comprised of many distinct families, including matrix metalloproteinases (MMPs) and the closely related a disintegrin and metalloproteinases (ADAMs) [13,14]. Metalloproteinases are capable of processing many extracellular proteins, including inter-MVEC junctional proteins, and can thereby disrupt MVEC barrier function and increase microvascular permeability [15]. Endothelial cell (EC) intercellular junctions, which are comprised of adherens and tight junctions, are dynamic structures located between adjacent EC that are critical to the formation of the EC barrier [7,16]. Adherens junctions are composed primarily of vascular endothelial (VE)–cadherin and tight junctions include occludins and claudins [16]. The relative proportions of different intercellular junctions varies between vascular beds, with adherens junctions accounting for 80% of the intercellular junctions within the lungs [4,17].

Metalloproteinases are regulated via gene expression, compartmentalization, pro-enzyme activation, and enzyme inactivation through the action of specific inhibitors, the TIMPs [18,19,20]. There are four TIMPs (TIMP1–4) in humans, each with the ability to inhibit the various metalloproteinases with different specificity [19,20]. For example, TIMP1 does not appear to be able to regulate MT–MMPs [21,22]. In addition, ADAMs and a disintegrin and metalloproteinases with thrombospondin motifs (ADAMTS) appear to be primarily inhibited by TIMP3 [21]. Some TIMPs have also been found to form a complex between an active MMP and latent pro-MMP, leading to activation of the latent MMP. This has been demonstrated extensively with TIMP2, which binds active MMP14 and latent MMP2, and facilitates MMP2 activation [20]. By regulating metalloproteinase activity, TIMPs may facilitate maintenance of MVEC barrier function.

Changes in metalloproteinase and TIMP expression in response to pro-inflammatory cytokines have been characterized in different types of EC [23,24,25,26,27,28,29]; however, data in MVEC, specifically PMVEC under septic conditions, is limited [30]. Moreover, the potential role of the balance between the family of metalloproteinases and TIMPs in regulating septic PMVEC barrier dysfunction has not been examined. We hypothesized that sepsis leads to a disruption of the balance between PMVEC-specific metalloproteinases and TIMPs, leading to increased metalloproteinase activity and, ultimately, septic murine PMVEC barrier dysfunction.

## 2. Results

### 2.1. Changes in Timp Expression by PMVEC under Septic Conditions Depends on the Specific Timp and the Septic Stimulus

**Figure 1:**
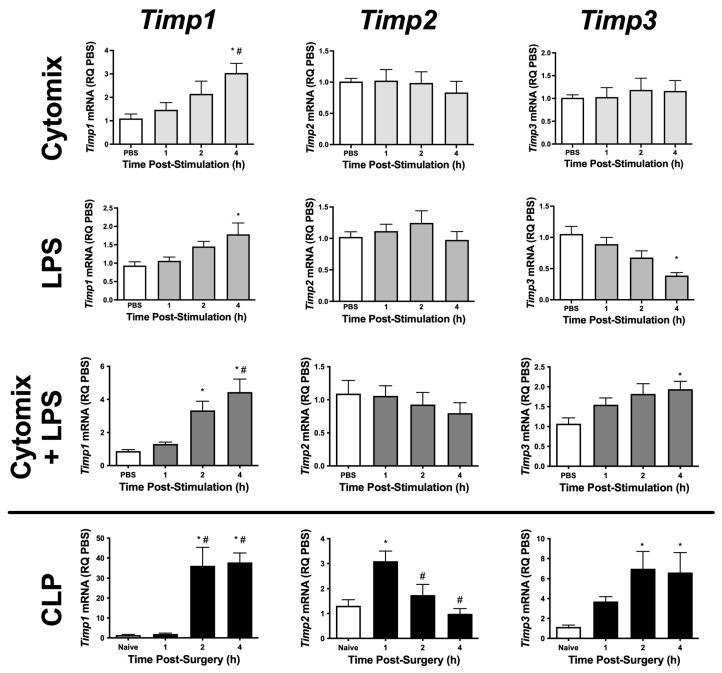
mRNA expression of various *Timps* is differentially altered in PMVEC under septic conditions in vitro and ex vivo. Cytomix-stimulated PMVEC had an increase in *Timp1* expression over 4 h vs. PBS control (data expressed as relative quantity [RQ] of PBS), as did lipopolysaccharide (LPS)-stimulated PMVEC, with a greater increase following cytomix + LPS-stimulation. PMVEC expression of *Timp2* was unaffected by stimulation with cytomix, LPS, or the combination of cytomix and LPS. PMVEC expression of *Timp3* was unaffected by cytomix, decreased with LPS, but paradoxically increased with the combination of cytomix and LPS. PMVEC from cecal ligation and perforation (CLP)-septic mice had an increase in *Timp1*, *Timp2*, and *Timp3* expression vs. PMVEC from naïve mice (data expressed as RQ of naïve) at different time points. *Timp4* was expressed at only low levels under all conditions. * *p* < 0.05 vs. PBS and # *p* < 0.05 vs. 1 h, One-Way ANOVA followed by a Bonferroni post-hoc test, n = 5–8.

To begin to understand the role of TIMPs and metalloproteinases in PMVEC barrier function, we initially examined PMVEC-specific *Timp* mRNA expression in multiple septic models. *Timp1–3* appear to be expressed at high levels in PMVEC under basal conditions; however, *Timp4* was expressed at very low levels (Table 1). PMVEC stimulated with cytomix had significantly increased *Timp1* mRNA expression by 4 h post-stimulation but there were no changes in *Timp2* and -*3* mRNA expression (Figure 1). LPS-stimulation of PMVEC significantly increased *Timp1* mRNA expression by 4 h post-stimulation, had no effect on *Timp2* mRNA expression, and significantly reduced *Timp3* mRNA expression by 4 h post-stimulation (Figure 1). The combination of cytomix and LPS resulted in significant increases in *Timp1* and -*3* mRNA expression as early as 2 h post-stimulation (*Timp1*), and this increase persisted at 4 h; however, no effect was observed on *Timp2* mRNA expression (Figure 1).

To further explore sepsis-induced changes in PMVEC-specific *Timp* expression in vivo, we isolated PMVEC from healthy and septic mice. PMVEC isolated from CLP-septic mice had significantly increased *Timp1* and -*3* mRNA expression as early as 2 h post-CLP vs. PMVEC isolated from naïve mice, and this increase remained significant at 4 h post-CLP. *Timp2* mRNA expression was significantly increased by 1 h post-CLP, but recovered to basal levels by 2 h post-CLP (Figure 1). Collectively, these studies demonstrate that in vitro, individual stimulations (i.e., cytomix or LPS alone) caused significant changes in *Timp* expression; however, the combined stimulation of cytomix and LPS in vitro appeared to be more representative of what was observed in vivo. Based on these findings, all subsequent studies were conducted using the combination of cytomix and LPS as an in vitro model of sepsis.

### 2.2. Altered Metalloproteinase Expression by PMVEC under Septic Conditions Is Dependent on the Specific Metalloproteinase Being Examined

**Figure 2:**
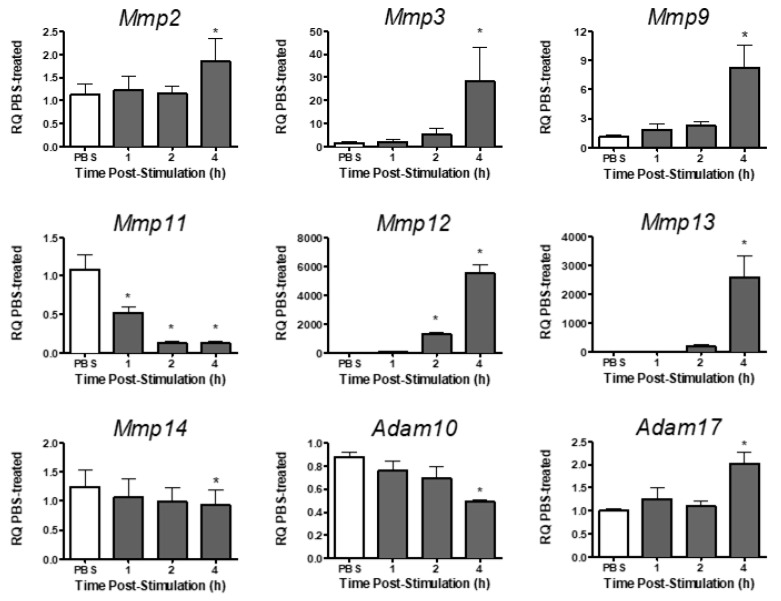
mRNA expression of various metalloproteinases is differentially altered in PMVEC in vitro under septic conditions. Cytomix + LPS-stimulated PMVEC had an increase in *Mmp2*, *-3*, *-9*, *-12*, *-13* and *Adam17* expression and a decrease in *Mmp11*, *-14,* and *Adam10* over 4 h vs. PBS control (data expressed as RQ of PBS). * *p* < 0.05 vs. PBS, One-Way ANOVA followed by a Bonferroni post-hoc test, n = 5–8.

Analysis of metalloproteinase expression in PMVEC revealed varying levels of expression for different metalloproteinases under basal conditions (Table 2). Under basal conditions, *Mmp2*, *-3*, *-9*, *-11*, *-13,* and *-14*, and *Adam10* and *-17* were expressed in PMVEC, whereas *Mmp12* was expressed at very low levels (Table 2). Following stimulation with both cytomix and LPS, *Mmp12* expression significantly increased at 2–4 h post-stimulation, and *Mmp2*, *-3*, *-9*, *-13,* and *Adam17* expression were significantly increased by 4 h post-stimulation (Figure 2). Expression of *Mmp11*, however, was significantly decreased as early as 1 h post-stimulation, and both *Mmp14* and *Adam10* mRNA expression were significantly decreased by 4 h post-stimulation (Figure 2).

### 2.3. Global Metalloproteinase Protein Activity Is Not Altered in PMVEC under Septic Conditions

**Figure 3:**
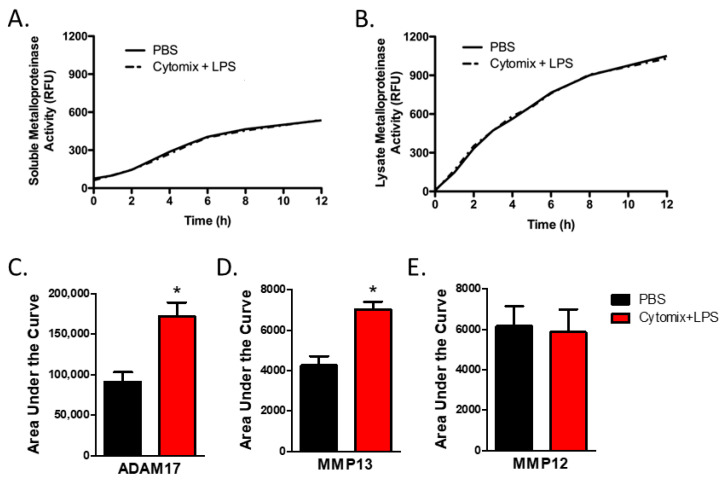
PMVEC-derived specific metalloproteinase activity is increased under septic conditions (cytomix + LPS). (**A**). PMVEC-derived total soluble metalloproteinase activity is not altered under septic conditions. Representative graph of metalloproteinase activity (indicated by increase in relative fluorescence over time) in the conditioned media of PMVEC under basal and septic conditions. n = 6. (**B**). Total metalloproteinase activity in PMVEC lysate is similarly not altered under septic conditions. n = 4. (**C**). MMP13 activity is significantly increased in PMVEC under septic vs. basal (PBS) conditions. * *p* < 0.05 vs. PBS, paired *t*-test, n = 8. (**D**). ADAM17 activity is significantly increased in PMVEC following septic vs. PBS stimulation. * *p* < 0.05 vs. PBS, paired *t*-test, n = 14. (**E**). MMP12 activity was not significantly altered in septic PMVEC. n = 11.

As analysis of mRNA expression is not necessarily reflective of changes in protein levels or, more relevantly, changes in the balance in the activities of metalloproteinases and TIMPs, we next examined metalloproteinase activity. To examine the activity of extracellular secreted soluble metalloproteinases, we assessed total metalloproteinase activity within the conditioned media from PMVEC under basal conditions and after 4 h of septic (cytomix and LPS) stimulation. Compared to PBS control, global soluble metalloproteinase activity was not significantly altered under septic conditions (Figure 3A).

While soluble metalloproteinases are detected in the conditioned media, many metalloproteinases, including ADAMs, are found on the cell membrane [13,14]. Further, soluble metalloproteinases expressed by PMVEC but not yet secreted, or bound to the external cell surface through interaction with cell surface receptors such as integrins, may not be captured by soluble activity assays as they are still associated with the cellular compartment. Thus, we next investigated global metalloproteinase activity within the PMVEC lysate. Compared to PBS control, total cellular metalloproteinase activity was not significantly altered under septic conditions (Figure 3B).

### 2.4. Specific Metalloproteinase Activity Is Altered in PMVEC under Septic Conditions

PMVEC expression of *Adam17*, a metalloproteinase known to be associated with the cell membrane, was significantly increased under septic conditions in vitro, as shown above. While total metalloproteinase activity was not altered under septic conditions, analysis of ADAM17 activity within the PMVEC lysate revealed that ADAM17 activity was significantly increased following the combined stimulation of cytomix and LPS vs. PBS control (Figure 3C). Analysis of the activities of individual MMPs in conditioned media revealed that the activity of MMP13 was significantly increased by cytomix and LPS stimulation vs. PBS control (Figure 3C). However, while PMVEC expression of *Mmp12* was significantly increased following in vitro stimulation with cytomix and LPS, activity of MMP12 did not appear to increase (Figure 3C).

### 2.5. Inhibition of Metalloproteinase Activity Reduces PMVEC Barrier Dysfunction under Septic Conditions

**Figure 4:**
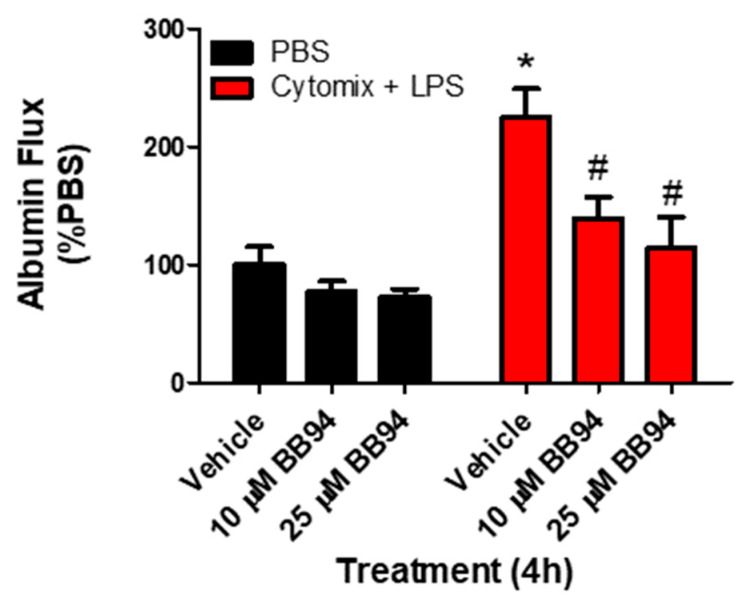
Inhibition of metalloproteinase activity by global metalloproteinase inhibitors reduces septic wild-type PMVEC barrier dysfunction. Trans-PMVEC albumin flux is significantly increased in vehicle treated PMVEC under septic vs. basal conditions. Treatment of PMVEC with BB94, a global metalloproteinase inhibitor, reduced the septic albumin flux. * *p* < 0.05 vs. respective PBS, # *p* < 0.05 vs. respective Vehicle, Two-Way ANOVA followed by a Bonferroni post-hoc test, n = 4.

**Figure 5:**
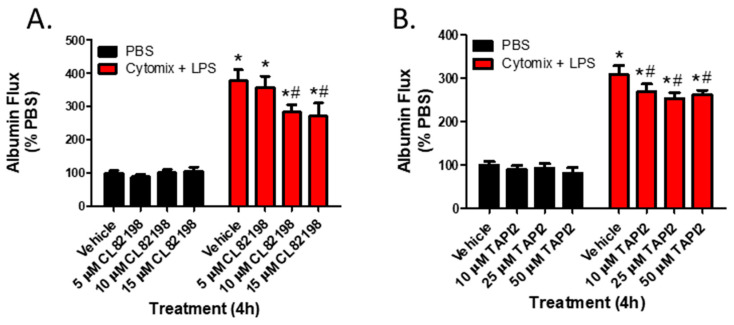
Inhibition of metalloproteinase activity by specific metalloproteinase inhibitors reduces septic wild-type PMVEC barrier dysfunction. (**A**). The septic increase in trans-PMVEC albumin flux is significantly reduced by pre-treatment with CL82198, an MMP13 inhibitor. * *p* < 0.05 vs. respective PBS, # *p* < 0.05 vs. respective Vehicle, Two-Way ANOVA followed by a Bonferroni post-hoc test, n = 3. (**B**). Similarly, the septic increase in albumin flux is significantly reduced following treatment with TAPI2, an ADAM17 inhibitor. * *p* < 0.05 vs. respective PBS, # *p* < 0.05 vs. respective Vehicle, Two-Way ANOVA followed by a Bonferroni post-hoc test, n = 7.

To examine whether the observed changes in metalloproteinase mRNA expression and subsequent increases in metalloproteinase activity, specifically MMP13 and ADAM17, were responsible for septic PMVEC barrier dysfunction in vitro, we next examined PMVEC barrier dysfunction in the presence of metalloproteinase inhibitors. PMVEC stimulation with cytomix and LPS for 4 h was associated with a significant increase in PMVEC permeability as indicated by increased albumin flux across the PMVEC monolayer. Batimastat (BB94), a broad-spectrum metalloproteinase inhibitor, significantly reduced albumin flux vs. DMSO control at concentrations of 10 and 25 μM (Figure 4). Moreover, inhibition of MMP13 with CL82198 also led to significantly reduced albumin flux at 10 μM and 15 µM (Figure 5). Treatment with TAPI2, an ADAM17 inhibitor, slightly reduced albumin flux with a significant reduction observed at 10, 25, and 50 µM (Figure 5).

### 2.6. Global and Specific Metalloproteinase Inhibitors Attenuate Septic Disruption in Inter-PMVEC Junctional Protein Surface Localization

**Figure 6:**
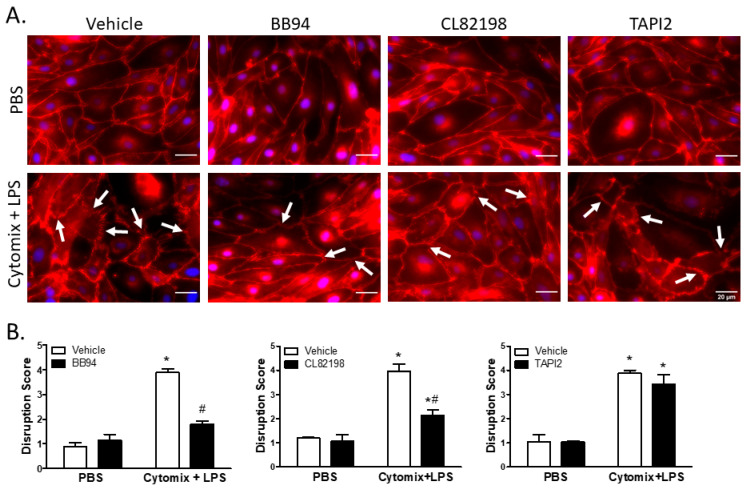
Septic disruption of VE–cadherin inter-PMVEC localization appears to be due to metalloproteinase activity. (**A**). In PMVEC under basal conditions (PBS), VE–cadherin staining (red) exhibited a linear circumferential pattern regardless of treatment with metalloproteinase inhibitors (BB94, CL82198, or TAPI2) vs. vehicle. Stimulation of PMVEC with cytomix + LPS led to disruption of PMVEC surface VE–cadherin continuity (arrows), and treatment with BB94 (25 µM) or CL82198 (10 µM) appeared to rescue this septic PMVEC VE–cadherin disruption. However, treatment with TAPI2 (25 µM) did not rescue septic VE–cadherin disruption. (**B**). Quantification of PMVEC surface VE–cadherin disruption reveals the septic disruption is attenuated in the presence of metalloproteinase inhibitors. VE–cadherin disruption in septic PMVEC is significantly reduced by treatment with 25 µM BB94 (LEFT) or with 10 µM CL82198 (MIDDLE). Septic VE–cadherin disruption, however, is not affected by the presence of 25 µM TAPI2 (RIGHT). * *p* < 0.05 vs. respective PBS, # *p* < 0.05 vs. respective vehicle, Two-Way ANOVA followed by a Bonferroni post-hoc test, n = 3.

Under basal conditions, surface localization of VE–cadherin was continuous around PMVEC borders; however, PMVEC surface VE–cadherin localization became disrupted following treatment with cytomix and LPS (Figure 6). This septic disruption of VE–cadherin localization appeared to be rescued in PMVEC pre-treated with 25 µM BB94 (Figure 6). Moreover, treatment with CL82198 also appeared to attenuate septic VE–cadherin disruption; however, treatment with TAPI2 had no effect on PMVEC surface VE–cadherin disruption (Figure 6). Quantification of septic induced VE–cadherin disruption by reviewers blinded to treatment identified a significant VE–cadherin disruption following treatment with cytomix and LPS that was significantly diminished by treatment with BB94 and CL82198, but not by TAPI2 (Figure 6).

**Figure 7:**
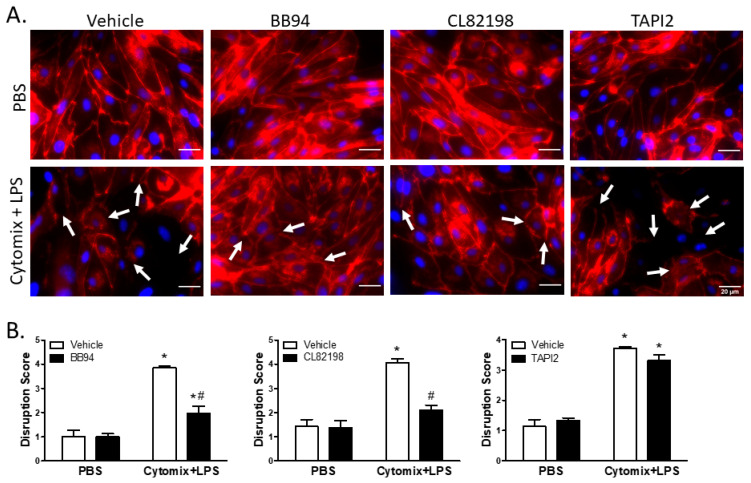
Septic disruption of claudin5 intercellular localization appears to be due to metalloproteinase activity. (**A**). In PMVEC under basal conditions (PBS), claudin5 staining (red) exhibited a linear circumferential pattern regardless of treatment with metalloproteinase inhibitors (BB94, CL82198, or TAPI2) vs. vehicle. Stimulation of PMVEC with cytomix + LPS led to disruption of dPMVEC surface claudin5 continuity (arrows), as well as reduced claudin5 abundance. Treatment with BB94 (25 µM) or CL82198 (10 µM) appeared to rescue this septic PMVEC claudin5 disruption, whereas treatment with TAPI2 (25 µM) did not rescue claudin5 disruption. (**B**). Quantification of PMVEC surface claudin5 disruption reveals that the septic disruption is attenuated in the presence of metalloproteinase inhibitors. claudin5 disruption in septic PMVEC is significantly reduced with 25 µM BB94 (LEFT) and 10 µM CL82198 (MIDDLE). Septic claudin5 disruption, however, is not reduced by the presence of 25 µM TAPI2 (RIGHT). * *p* < 0.05 vs. respective PBS, # *p* < 0.05 vs. respective vehicle, Two-Way ANOVA followed by a Bonferroni post-hoc test, n = 3.

Similar to VE–cadherin, surface localization of claudin5 appeared continuous around the cell borders of PMVEC under basal conditions; however, treatment with cytomix and LPS caused disruption of surface claudin5 as well as an overall reduction in claudin5 abundance (Figure 7). Treatment with BB94 appeared to rescue the septic disruption of claudin5 (Figure 7). Additionally, disruption of claudin5 appeared to be rescued by application of CL82198; however, TAPI2 had no effect on septic disruption of claudin5 (Figure 7). Quantification of images by reviewers blinded to treatment assignment demonstrated a significant claudin5 disruption following stimulation with cytomix and LPS (Figure 7). Moreover, inhibition of metalloproteinase activity by BB94 or CL82198 significantly reduced septic claudin5 disruption (Figure 7). Similar to VE–cadherin, the cytomix and LPS-induced disruption of claudin5 was not rescued by the application of TAPI2 (Figure 7).

## 3. Discussion

In this study, we tested the hypothesis that sepsis leads to a disruption of the balance between PMVEC-specific metalloproteinases and TIMPs, leading to increased metalloproteinase activity and, ultimately, septic murine PMVEC barrier dysfunction. PMVEC-dependent global metalloproteinase activity under septic conditions was similar to basal levels, potentially due to a corresponding increase in the expression of certain *Timps* along with the observed increases in specific metalloproteinase expression. Moreover, the activities of individual metalloproteinases, such as MMP13 and ADAM17, were significantly increased and associated with septic barrier disruption. Importantly, inhibition of metalloproteinase activity by synthetic metalloproteinase inhibitors reduced the disruption of the inter-PMVEC junctional proteins and the corresponding albumin leak under septic conditions. Overall, our data supports our hypothesis, and suggests a potentially important role for a disturbance in the balance between metalloproteinases and TIMPs in septic pulmonary microvascular barrier function. This is critically important, as the microvasculature in individual organs is an important aspect of the pathophysiology of septic organ dysfunction, with loss of barrier function often resulting in tissue edema and organ dysfunction [31,32].

There are potentially many mechanisms of septic PMVEC barrier dysfunction, including PMVEC retraction, apoptosis, and disruption of intercellular junctions [2,33,34,35,36]. Disruption of intercellular adherens junctions occurs through multiple mechanisms, including through direct proteolytic degradation of key junctional proteins by serine proteases, as well as potentially by metalloproteinases [37,38,39,40]. Within these studies, we first explored different in vitro conditions reflecting sepsis and compared it to the response obtained ex vivo in cells from septic animals. In comparison to cytomix or LPS treatment alone, the combination of cytomix and LPS treatment of PMVEC led to barrier dysfunction and changes in gene expression that were similar to those observed in PMVEC isolated from septic mice.

The decrease in *Timp3* mRNA expression in LPS-stimulated PMVEC is consistent with previous studies, in which TIMP3 expression (mRNA and protein) was significantly decreased in murine brain MVEC and PMVEC following treatment with IFNγ, IL1β, and TNFα [26,30]. Conversely, our finding that *Timp3* mRNA expression increased in PMVEC stimulated with both cytomix and LPS, similar to the observed increase in expression in CLP-septic PMVEC ex vivo, suggests that more intense septic stimulation is required to induce *Timp3* expression. Human recombinant IL1α/β, TNFα, and IFNγ act synergistically, and the combination of IFNγ with TNFα or IL1α/β led to a greater increase in permeability in HUVECs compared to either cytokine alone [41]. In human sepsis, as well as murine CLP-sepsis in vivo, both cytokines and LPS are present, collectively contributing to excessive inflammation and tissue/organ injury [42,43]. Therefore, an in vitro model using both cytokines and LPS may be more reflective of the in vivo conditions during sepsis, and may be a more biologically-relevant model than cytomix or LPS individually.

Importantly, TIMP expression appears to be differentially regulated under conditions of infection and organ injury [44,45,46,47]. Our observed increase in *Timp1* mRNA expression in septic PMVEC is consistent with previous studies in other vascular beds [26,27]. Moreover, TIMP1 levels in serum have been found to be increased in severe sepsis in humans [48,49]. Thus, these previous studies combined with the strong increase in PMVEC *Timp1* expression seen in our dual stimulation in vitro, as well as in PMVEC isolated from mice with CLP-sepsis in vivo, strongly suggest a potential role for PMVEC-specific TIMP1 in tissue injury and sepsis.

Another major finding from our study was that septic disruption of PMVEC intercellular junctions, including adherens and tight junctions, was associated with an increase in specific metalloproteinase expression, including *Mmp3*, *-12,* and *-13*, as well as *Adam17*. While changes in metalloproteinase expression do not always correlate with changes in activity, the observed septic increase in *Mmp13* expression was associated with an increase in MMP13 activity. Similarly, expression of *Adam17* was increased under septic conditions, which correlated with an observed increase in ADAM17 activity.

MMP13 has previously been found to be upregulated in preclinical models of sepsis; however, the source of MMP13 in these studies appeared to be epithelial cells and inflammatory cells [50,51]. Similar to our study, others have reported an upregulation of *Adam17* expression in murine brain ECs following stimulation with pro-inflammatory cytokines such as TNFα, IL1β, and IFNγ [25]. Additionally, LPS has been shown to increase the expression of *ADAM17* in human PMVEC [52]. Collectively, these studies support our findings that metalloproteinase expression by ECs is altered under pro-inflammatory and septic conditions. However, while ECs, including PMVEC, have previously been found to express multiple metalloproteinases, our study is the first to demonstrate a septic increase in PMVEC-specific MMP13.

Unlike MMP13, the septic increase in *Mmp12* gene expression in murine PMVEC was not associated with a corresponding increase in MMP12 activity. MMP12 has been found previously to have functions that do not require enzymatic activity [53]. For example, in both viral-infected human lung myocardial cells and mouse cardiac fibroblasts, MMP12 has been found to translocate to the nucleus and function as a transcription factor [53]. Thus, it is possible that MMP12 may have other, non-enzymatic functions in PMVEC under septic conditions.

Interestingly, it is unclear why total metalloproteinase activity did not appear to change under septic conditions while the activity of specific metalloproteinases, MMP13 and ADAM17, increased. Our studies do, however, clearly demonstrate the key role of metalloproteinase activity in driving PMVEC barrier dysfunction as global inhibition of metalloproteinases with a synthetic metalloproteinase inhibitor attenuates septic PMVEC barrier dysfunction. Specifically, BB94, a potent broad-spectrum inhibitor for MMP1, -2, -3, -7, -9, -14, and ADAM17 [54], reduced albumin and dextran flux by 50% in cytomix- and LPS-stimulated PMVEC. This is consistent with the literature, as previous studies found that BB94 significantly inhibited alveolar–capillary leak in rats, as well as reduced blood–brain barrier permeability in rats [55,56,57].

We were also able to rescue PMVEC barrier function and reduce the septic increase in permeability by pre-treatment of PMVEC with metalloproteinase inhibitors that target specific metalloproteinases. Because both specific metalloproteinase inhibitors, TAPI2 and CL82198, were able to reduce the septic increase in PMVEC permeability, both ADAM17 and MMP13 may be critical mediators of septic PMVEC barrier dysfunction. Dreymueller et al. have shown that permeability was increased in human MVEC due to LPS stimulation; this septic permeability was attenuated by the application of GW280264X, which is an ADAM10 and -17 inhibitor, suggesting that ADAM17 may play a role in septic barrier dysfunction and increased permeability [52]. Further, LPS-induced permeability in the gut was attenuated in *Mmp13^−/−^* mice, compared to the *Mmp13^+/+^* mice [50]. This supports a role for MMP13 in mediating septic barrier dysfunction.

Surprisingly, although we observed a reduction in septic permeability with inhibition of ADAM17 in murine PMVEC, septic VE–cadherin and claudin5 disruption were not rescued by the inhibition of ADAM17 activity. Instead, inhibition of MMP13 activity rescued the VE–cadherin and claudin5 disruption in murine PMVEC. This suggests that MMP13 may be involved in adherens and tight junctional protein degradation in mouse PMVEC. Our data are supported by the previous studies from Lu et al., who observed that recombinant MMP13 was able to degrade the tight junctional protein zona occludens 1 (ZO1), leading to increased permeability in rat brain ECs [58].

Collectively, our study provides novel data on the impact of specific metalloproteinases, especially MMP13, on septic PMVEC barrier dysfunction. There are some limitations, however, in our study design. Due to technological limitations, we were not able to replicate a flow model that PMVEC would encounter in their native conditions (i.e., hemodynamic factors including shear stress and variation in vessel size). However, our initial in vivo study of PMVEC gene expression and our use of multiple in vitro models of septic stimulation created various environments for the endothelium to respond to, one of which appears to be a strong representation of the septic environment in vivo based on our ex vivo septic PMVEC expression analysis. Overall, we believe that the use of multiple models strengthened our in vitro analysis, and was also supported by direct ex vivo study of PMVEC isolated from septic mice.

Additionally, while TNFα, IL1β, and IFNγ are seen in human sepsis, they are considered to be pro-inflammatory cytokines, and not sepsis-specific. Previous studies have identified significant increases in several other cytokines not included in our “cytomix”, including monocyte chemotactic protein 1, IL6 and -8, granulocyte colony-stimulating factor, hepatocyte growth factor, and macrophage inflammatory protein 1β, in plasma from patients with severe sepsis [59]. Lastly, all experiments were conducted using murine cells, and as such, human cells must be employed in future studies to investigate this endogenous metalloproteinase/TIMP-dependent mechanism protecting against septic PMVEC barrier dysfunction. These studies would provide an opportunity to assess in human PMVEC the molecular mechanisms regulating normal and septic microvascular permeability that we have identified in murine PMVEC, which will give further insight regarding the physiological relevance of these findings, as well as provide strong clinical relevance.

In conclusion, we have identified a septic dysregulation in the metalloproteinase/TIMP balance, leading to increased metalloproteinase activity associated with septic PMVEC barrier dysfunction. This septic PMVEC barrier dysfunction is driven by a metalloproteinase-dependent mechanism, and is potentially mediated through ADAM17 and MMP13, resulting in increased microvascular permeability due to adherens and tight junctional protein degradation. Our results suggest that inhibition of metalloproteinase activity could be very important in rescuing the PMVEC barrier function during sepsis. Moreover, our results suggest that PMVEC-specific TIMPs may support pulmonary microvascular endothelial barrier function via the direct inhibition of metalloproteinase activity. A better understanding of the TIMP-mediated endogenous protective mechanism as well as the protection mediated by exogenous synthetic metalloproteinase inhibitors against septic PMVEC barrier dysfunction would support new therapeutic interventions in human sepsis and related septic organ dysfunction.

## 4. Materials and Methods

### 4.1. Pulmonary Microvascular Endothelial Cell (PMVEC) Isolation for In Vitro Analysis

Murine PMVEC were isolated from the lungs of healthy male C57BL/6 mice (8–12 weeks of age) and cultured for use in all in vitro experiments, as previously described [30,60,61]. In brief, following lung isolation, lung tissue was finely minced and digested using 0.3% collagenase (Worthington Biochemical Corp., Lakewood, NJ, USA) in Hank’s Balanced Salt Solution (HBSS, Invitrogen, Carlsbad, CA, USA). Following filtration through a 100 μm pore mesh sieve, cells were incubated with magnetic microbeads (Dynabeads M-450 sheep anti-rat IgG, Dynal Biotech Inc., Lake Success, NY, USA) coupled to anti-platelet endothelial cell adhesion molecule (PECAM; CD31) antibodies (Rat anti-mouse CD31 monoclonal antibody, BD Pharmingen, Franklin Lakes, NJ, USA). Microbead-bound PMVEC were magnetically captured (MPC magnet, Dynal Biotech Inc.), and subsequently washed and suspended in growth medium (Dulbecco’s modified Eagle’s medium [DMEM] supplemented with 20% heat inactivated fetal bovine serum [FBS], 1% Penicillin/Streptomycin [10,000 U/mL], and 2% [4-2[2-hydroxyetyl]-1-piperazineethanesulfonic acid] [HEPES] buffer [1M]; Invitrogen). PMVEC were then seeded into a 1% gelatin-coated cell culture flask and incubated at 37 °C with 5% carbon dioxide (CO_2_). Cells were assessed weekly to ensure appropriate morphology, and once approximately 90% confluent, cells were stained with fluorescent acetylated low-density lipoprotein (LDL) (Biomedical Technologies, Stoughton, MA, USA) and assessed by immunofluorescence, as well as stained with fluorescently-labelled antibodies against endothelial cell markers (anti-CD31, CD34, CD146 and CD202; VWR International, Mississauga, ON, Canada) and assessed by flow cytometry to ensure purity. Collectively, these processes resulted in 99% PMVEC culture homogeneity. PMVEC were then grown in supplemented DMEM growth medium and incubated at 37 °C with 5% CO_2_ until the cell monolayer reached confluence. PMVEC at passages 5–12 were used for all experiments.

### 4.2. PMVEC Isolation from Healthy and Septic Mice for Ex Vivo Analysis

PMVEC were isolated from the lung tissue of healthy (naïve) and septic (cecal ligation and perforation [CLP] model of sepsis for 1, 2, or 4 h) mice, as previously described [2,30]. PMVEC were isolated using the Mouse Lung Dissociation Kit (Miltenyi Biotec, Auburn, CA, USA) as per the manufacturer instructions. Briefly, the pulmonary vasculature was flushed with 10 mL saline and the lungs isolated. Lungs were then physically disrupted using the gentleMACS^TM^ Dissociator (Miltenyi), followed by chemical digestion via Collagenase D and DNAse A (Miltenyi) for 30 min at 37 °C. The whole lung digest was strained through 100 and 70 µm cell strainers (Miltenyi) and resuspended in DMEM supplemented with 0.5% bovine serum albumin (BSA) and 0.5 M EDTA (pH 8.0). Once isolated, the whole lung cell suspension was incubated with CD45 mouse MicroBeads (Miltenyi) for 15 min at 4 °C, and then CD45^+^ cells were magnetically separated using LS columns (Miltenyi). The CD45^−^ cells were then incubated with CD31 mouse MicroBeads (Miltenyi) for 15 min at 4 °C, and the CD45^−^/CD31^+^ cells magnetically isolated using new LS columns. These cells were spun at 300× *g* for 10 min at 4 °C, resuspended in phosphate buffered saline (PBS) with RNAse inhibitor (4 U/µL, Qiagen, Hilden, Germany), and placed at −80 °C for storage until future analysis by quantitative real-time polymerase chain reaction (qRT-PCR). As above, purity of the isolated CD45^−^/CD31^+^ cells was confirmed by flow cytometry using fluorescently-labelled antibodies against endothelial cell markers (CD31, CD34, CD146 and CD202b).

### 4.3. Assessment of PMVEC-Specific Timp and Metalloproteinase Expression

Expression levels of *Timp1–4* were examined in PMVEC in vitro and ex vivo by qRT-PCR. For in vitro analysis, cells were cultured in 6-well cell culture plates coated with 1% gelatin. Once confluent, PMVEC were treated with PBS (vehicle control), cytomix (an equimolar solution of TNFα, IL1β, and IFNγ, 30 ng/mL, PeproTech, Rocky Hill, NJ, USA), LPS (10 μg/mL, 0111:B4 serotype, Sigma, Oakville, ON, Canada), or a combination of both cytomix and LPS (30 ng/mL, 10 μg/mL, respectively) for 1, 2, and 4 h. Following stimulation, cells were lysed and RNA isolated using the RNeasy Mini Kit following the manufacturer’s directions (Qiagen). Briefly, 200 μL RLT buffer containing 2 μL beta-mercaptoethanol was added to each well, wells scraped, and cell lysate collected into QiaShredder tubes (Qiagen). Samples were spun at 14,000× *g* for 2 min, and flow-through collected into RNeasy spin columns, washed with a series of buffers, and RNA eluted from the columns by water.

For ex vivo analysis, RNA was isolated using the RNeasy Micro Kit (Qiagen) from PMVEC isolated from naïve and septic mice as described above. Briefly, cells were spun at 14 000× *g* for 2 min, and subsequently run through a genomic column for 1 min at 10,000× *g*. Flow-through was collected and washed, and the RNA eluted from the columns. RNA purity and concentration were determined by analysis with the NanoDrop 1000 spectrophotometer (Thermo Scientific, Waltham, MA, USA). Isolated RNA (2 μg) was reverse transcribed using a high-capacity cDNA reverse transcription kit (Invitrogen) according to manufacturer’s instructions.

Additionally, expression of *Mmp2*, -*3*, -*9*, -*11*, -*12*, -*13*, -*14*, *Adam10*, and -*17* were examined in PMVEC in vitro. Based on the data from the analysis of PMVEC-specific *Timp* expression, PMVEC were treated with PBS or with both cytomix and LPS (30 ng/mL, 10 μg/mL, respectively) for 1, 2, and 4 h to mimic septic conditions.

Gene expression was assessed using TaqMan Gene Expression Assays from Applied Biosystems (Invitrogen) and the CFX96 Real Time System (BioRad Laboratories Inc., Hercules, CA, USA). Cycling conditions included an initial enzyme activation step at 95 °C for 10 min, followed by 50 cycles of denaturation at 95 °C for 10 s, and annealing and extension at 60 °C for 1 min. Hypoxanthine-guanine phosphoribosyltransferase (Hprt) was used to normalize expression of genes of interest (Invitrogen). qRT-PCR generated the cycle threshold (C_T_) value for each gene, and this value was then used to determine gene expression relative to control PMVEC (PBS-treated or isolated from naïve/sham mice). ΔC_T_ was the normalization of the gene of interest to the housekeeping gene within a specific sample. ΔΔC_T_ was the normalization of a specific sample to the control sample. Finally, the relative quantity (RQ) was the fold change in expression of a specific sample relative to the control sample. RQ was determined by the following equation: RQ = 2^−ΔΔCt^.

### 4.4. Assessment of PMVEC Metalloproteinase Activity

Male PMVEC were grown to confluence in 6-well cell culture plates coated with 1% gelatin and treated for 4 h with both cytomix and LPS (30 ng/mL and 10 μg/mL) vs. PBS (vehicle control). Following stimulation, the conditioned media were collected, concentrated using centrifugal filter units (Amicon Ultra-4, 10 K, Milipore Sigma, St. Louis, MO, USA) by centrifugation (2000× *g* for 30 min), and placed at −80 °C for storage. Cells were rinsed with PBS and then lysed by directly adding lysis buffer (1X CellLytic extraction buffer, Sigma-Aldrich) into each well. Cells were then placed on ice for 20 min, after which the cell lysate was collected, spun at 20,000× *g* for 15 min, and the supernatant drawn off, aliquoted, and frozen at −80 °C.

Global metalloproteinase activity in the conditioned media and cell lysate was analyzed using the P126 OmniMMP fluorogenic substrate as per the manufacturer instructions (Enzo Life Sciences, Burlington, ON, USA). Briefly, 85 μL of conditioned media or 25 μL of cell lysate from each sample was added to each well of a clear-bottom 96-well black microplate (VWR). Fluorescently-labelled P126 substrate and assay buffer were then added to each well, including positive controls. Wells containing the reference peptide, P127 (Enzo Life Sciences), were used as positive controls, and wells containing sample and assay buffer but no P126 substrate were used as negative controls for background fluorescence. Fluorescence was measured (Excitation peak wavelength: 328 nm [320–340 nm]; Emission peak wavelength: 393 nm [393–405 nm]) using a Victor3 multilabel fluorescence microplate reader (Wallac) at 0, 3, 5, 7, 10, 20, and 30 min, and 1, 2, 3, 4, 5, 21, and 24 h. The plate was incubated at 37 °C with 5% CO_2_ between readings.

ADAM17 activity was assessed in the cell lysate using the Sensolyte 520 ADAM17 Activity Assay Kit as per the manufacturer instructions (AnaSpec, Fremont, CA, USA). Briefly, 50 μL of cell lysate from each sample was added to each well of a clear-bottom 96-well black microplate (VWR). Fluorescently-labelled substrate and assay buffer were added to each sample, along with positive and negative controls. Wells containing the substrate and assay buffer but no sample were used as negative controls for background fluorescence. The fluorescence was measured (Excitation peak wavelength: 490 nm; Emission peak wavelength: 520 nm) every 30 min for 2 h, and then every 60 min for 3–12 h. The plate was incubated at 37 °C with 5% CO_2_ between readings.

MMP12 and -13 activity was assessed in the conditioned media using the SensoLyte 520 MMP12 (Anaspec) or MMP13 Activity Assay Kit (Anaspec), as per the manufacturer instructions. Briefly, 45 μL of conditioned media from each sample were added to each well of a clear-bottom 96-well black microplate. To activate pro-MMP12 and pro-MMP13, 4-aminophenylmercuric acetate (APMA) was added to each well and incubated at 37 °C with 5% CO_2_. Fluorescently-labelled substrate and assay buffer were added to each sample, and fluorescence was measured as described above. MMP12 and -13 activity were assessed as above every 30 min for 2 h, and then every 60 min for 12–24 h. Changes in fluorescence over time were graphed, and area under the curve determined for statistical analysis.

### 4.5. Assessment of PMVEC Barrier Function following PMVEC Stimulation

PMVEC were seeded at a cell density of 5 × 10^4^ cells/insert on 1% gelatin-coated transwell cell–culture inserts (3.0 μm pore, VWR). PMVEC were cultured for 5–7 days to allow a stable monolayer to form, and then baseline PMVEC barrier permeability was comprehensively assessed using transendothelial electrical resistance (TEER) and Evans blue (EB)-labelled albumin flux, which is a clinically-relevant marker of both paracellular and transcellular permeability to large molecules. TEER, which was only used to determine when a PMVEC monolayer was established, was measured across PMVEC monolayers by placing individual cell–culture inserts into the Endohm chamber (World Precision Instruments, Sarasota, FL, USA) in order to measure the electrical resistance in Ohms (Ω) using the EVOM2 Endothelial Voltohmmeter (World Precision Instruments). TEER across empty individual inserts not containing PMVEC was used as a control to account for background resistance from the cell–culture inserts, and this value was subtracted from the TEER measurements obtained from inserts containing PMVEC. EB-labelled albumin was added directly to the upper chamber of the transwell insert containing the PMVEC monolayer, while the same concentration of unlabelled albumin was added to the lower chamber to control for osmotic pressure. Following 1 h, the transwell inserts were removed, and the conditioned media of the lower chamber were collected. Absorbance of the conditioned medium was measured (A_620_) with the iMark™ Microplate Reader (BioRad Laboratories Inc.). Background absorbance was measured in EB-albumin-free media.

We have previously reported the effects of cytomix stimulation (30 ng/mL) on TEER and trans-PMVEC macromolecular leak in PMVEC monolayers cultured on transwell cell-culture inserts [30,62]. These studies demonstrated that maximal trans-PMVEC EB-labelled albumin leak occurred 4 h following stimulation with cytomix [30]. To assess the effects of global metalloproteinase inhibitors on PMVEC barrier dysfunction under septic conditions, PMVEC were grown to confluence on cell–culture inserts as described above, and permeability assessed by EB-labelled albumin following a 4 h stimulation with both cytomix (30 ng/mL) and LPS (10 μg/mL) vs. PBS (vehicle control) in the presence of vehicle control (dimethyl sulfoxide [DMSO] or 1 of the following synthetic metalloproteinase inhibitors: (BB94 [10, 25 μM, EMD Millipore, Burlington, MA, USA], TAPI2 [10, 25, 50 μM, EMD Millipore], or CL82198 hydrochloride [5, 10, 15 μM, TOCRIS Bio-techne, Oakville, ON, Canada]). Cells were treated with metalloproteinase inhibitors or vehicle controls 16 h prior to stimulation with PBS or cytomix and LPS, and further incubated at 37 °C with 5% CO_2_.

### 4.6. Assessment of Cell–Cell Junctional Protein Localization by Immunohistochemistry

PMVEC were cultured in 1% gelatin-coated 24-well plates. Once a confluent monolayer was formed, PMVEC were treated with DMSO, 25 µM BB94, 25 µM TAPI2, or 10 µM CL82198 for 16 h (NOTE: Concentrations of inhibitors were based on the peak inhibition of leak observed in EB-albumin leak studies). Cells were stimulated with either cytomix (30 ng/mL) and LPS (10 µg/mL) or PBS (vehicle control) for 4 h, and fixed with 100% methanol. Following the three washes with PBS, cells were permeabilized with 0.1% Triton X-100 detergent (VWR), blocked with 3% BSA in PBS, and incubated with primary antibodies against VE–cadherin (rabbit polyclonal, 1:100 dilution, Abcam, Cambridge, MA, USA) or claudin5 (rabbit monoclonal, 1:100 dilution, Abcam) for 2 h. Following the three washes with PBS, cells were incubated with red-fluorescent Alexa Fluor^TM^ 594 secondary antibody (goat anti-rabbit IgG (H+L), 1:500 dilution, Invitrogen) for 1 h. Cell nuclei were stained with Hoechst 33342 in PBS (1:5000 dilution, Invitrogen). Localization of VE–cadherin and claudin5 was then examined by fluorescent microscopy (Zeiss Axiovert 200M Inverted Microscope; Carl Zeiss Canada Ltd., Toronto, ON, Canada). Cells stained with secondary antibody only were used as a negative control, and exposure times were kept constant for all subsequent images.

A blind study was conducted to quantify the surface localization of VE–cadherin or claudin5 disruption in images. Briefly, the amount of VE–cadherin or claudin5 disruption in each category was graded from 0 to 5 according to this score system (No disruption = 0, 20% = 1, 40% = 2, 60% = 3, 80% = 4, 100% = 5; Figures S1 and S2) by three reviewers. The mean of the data set of each category was used for statistical analysis.

### 4.7. Statistical Analysis

Differences between groups were assessed by *t*-test or a One-Way ANOVA with a Bonferroni post-hoc test for one independent variable, or by a Two-Way ANOVA with a Bonferroni post-hoc test for two independent variables using GraphPad Prism 6.2. Significance threshold was set at α = 0.05. Outliers were assessed using the Grubbs’ test.

## Figures and Tables

**Table 1:** Average basal *Timp* mRNA expression in isolated murine PMVEC in vitro (n = 22).

Gene Name	C_T_ Value
*Timp1*	28.4
*Timp2*	24.7
*Timp3*	25.2
*Timp4*	36.7
*Hprt*	25.6

Abbreviations: cycle threshold, C_T_; hypoxanthine-guanine phosphoribosyltransferase, *Hprt*; pulmonary microvascular endothelial cells, PMVEC; tissue inhibitors of metalloproteinases, *Timp.*

**Table 2:** Average basal metalloproteinase mRNA expression in isolated murine PMVEC in vitro (n = 22).

Gene Name	C_T_ Value
*Mmp2*	28.7
*Mmp3*	35.2
*Mmp9*	36.1
*Mmp11*	33.1
*Mmp12*	40
*Mmp13*	34.5
*Mmp14*	27.6
*Adam10*	25.6
*Adam17*	27.1
*Hprt*	25.6

Abbreviations: a disintegrin and metalloproteinase, *Adam*; cycle threshold, C_T_; hypoxanthine-guanine phosphoribosyltransferase, *Hprt*; matrix metalloproteinase, *Mmp*; pulmonary microvascular endothelial cells, PMVEC.

## Data Availability

All the data presented here are included in the manuscript. Further inquiries should be directed to the corresponding author.

## Supplementary Materials

The supporting information can be downloaded at: https://www.mdpi.com/article/10.3390/ijms24097875/s1.
