# Supplementary material for: Imbalance of Pulmonary Microvascular Endothelial Cell-Expression of Metalloproteinases and Their Endogenous Inhibitors Promotes Septic Barrier Dysfunction
Source: Int J Mol Sci. 2023 Apr 26;24(9):7875. doi: 10.3390/ijms24097875 (PMC10178398; doi:10.3390/ijms24097875)
Supplement: Supplementary file 1 [file ijms-24-07875-s001.zip › Supplemental Figures-Jayawardena et all-22-2-2023.pdf]

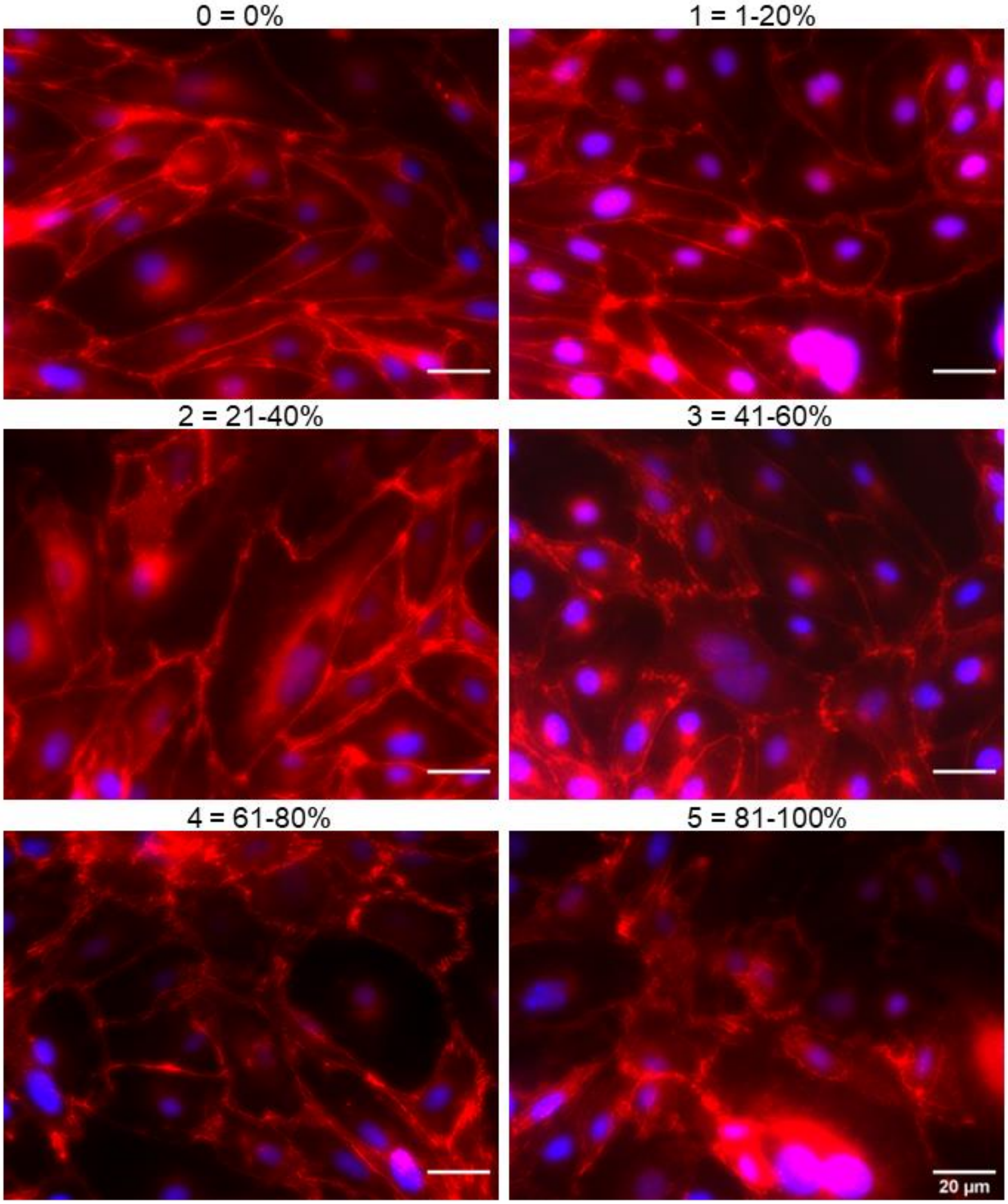

Figure S1. Scoring system of VE-cadherin disruption. To quantify the degree to which VE-cadherin was disrupted, a disruption score was created where the amount of VE-cadherin disruption was graded from 0 to 5 (0 = No disruption, 1-20% = 1, 21-40% = 2, 41-60% = 3, 61-80% = 4, 81-100% = 5). The disruption score was then determined by three independent reviewers blinded to treatment and condition and the mean of each category was used for statistical analysis.

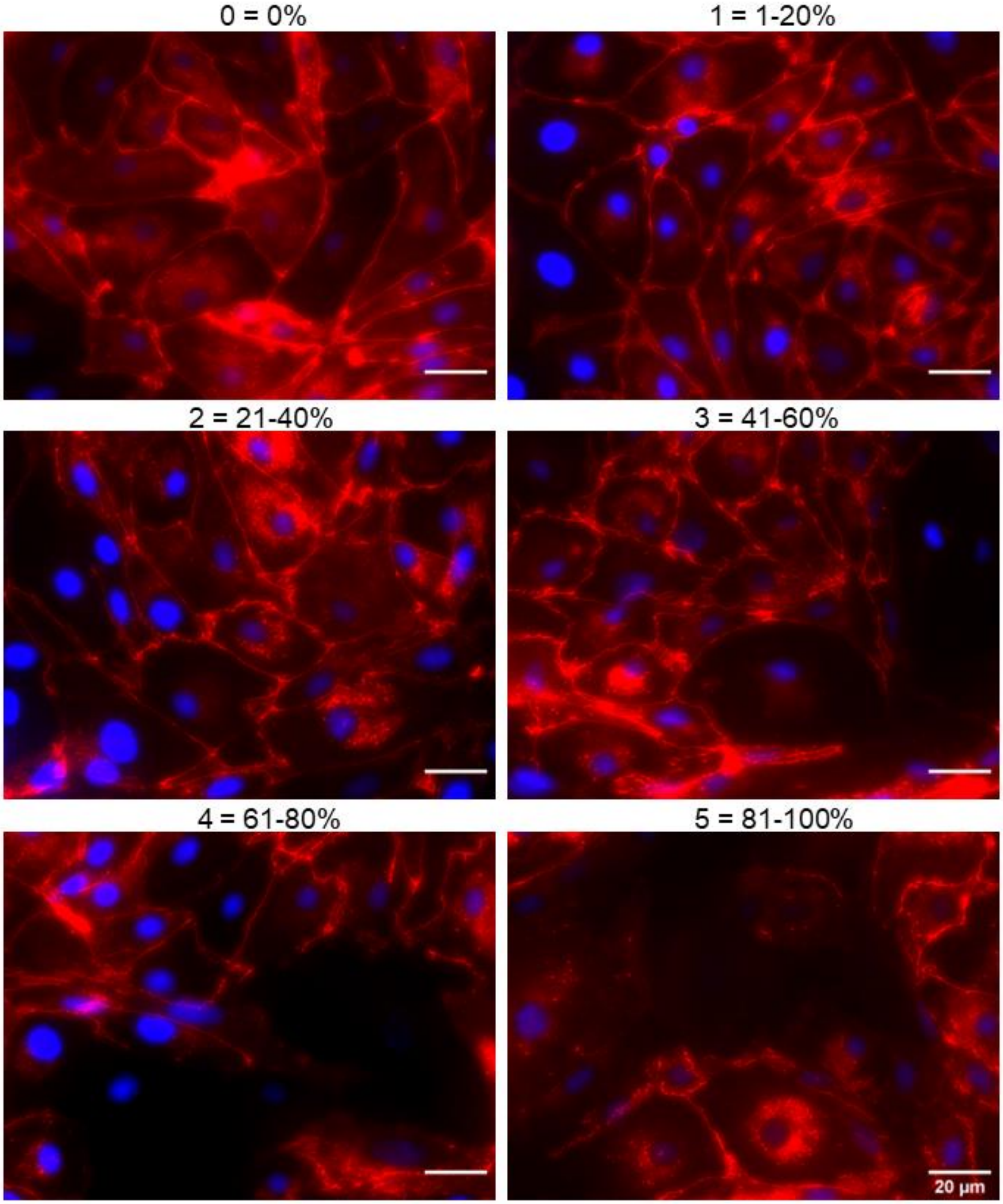

Figure S2. Scoring system of claudin5 disruption. To quantify the degree to which claudin5 was disrupted, a disruption score was created where the amount of claudin5 disruption was graded from 0 to 5 (0 = No disruption, 1-20% = 1, 21-40% = 2, 41-60% = 3, 61-80% = 4, 81-100% = 5). The disruption score was then determined by three independent reviewers blinded to treatment and condition and the mean of each category was used for statistical analysis.
